# Prostaglandin-D2 synthase localises to centrioles and primary cilium, and interacts with TOPORS, implicated in retinal ciliopathy

**DOI:** 10.1186/2046-2530-4-S1-P15

**Published:** 2015-07-13

**Authors:** B Czub, A Shah, P Kruczek, G Alfano, C Chakarova, S Bhattacharya

**Affiliations:** 1 Institute of Ophthalmology, University College London, London, UK

## Objective

Prostaglandin-D2 synthase (*PTGDS*; MIM#176803) is a novel protein-partner of TOPORS (*TOPORS*; MIM#609507), a ubiquitously expressed nuclear and ciliary protein, implicated in retinitis pigmentosa. This study investigated the localisation of PTGDS and its potential mechanism-of-association with TOPORS.

## Methods

Yeast two-hybrid screens, using TOPORS as bait, were performed against human retinal cDNA libraries. Validation and interaction-characterisation were performed in yeast, and by co-immunoprecipitation (co-IP) from HeLa cell extracts. Co-localisation studies were performed in hTERT-RPE1 cell line, and in murine retina cryo-sections. *PTGDS *expression was validated by RT-PCR.

## Results

Co-IP demonstrated PTGDS was found in endogenous protein complexes with TOPORS, whereas in yeast PTGDS interacted most strongly with TOPORS' residues 1-380, comprising the RING-domain conferring its E3-ubiquitin-ligase activity. PTGDS co-localised with TOPORS, and centriolar markers in dividing cells, and was observed at basal body and along ciliary axoneme in ciliated cells. In mouse retina PTGDS was observed in several cell layers, partly overlapping with TOPORS in the photoreceptor layer. In human retina, RT-PCR studies demonstrated expression of several *PTGDS *isoforms.

## Conclusion

PTGDS, a novel component of the primary cilium, could be involved in centriolar-ciliary homeostasis. This putative role of prostaglandin synthases, is additionally supported by independent findings on the role of prostaglandin-E2 in ciliogenesis. Results suggest TOPORS could regulate PTGDS levels at the cilium by marking it for degradation by the ubiquitin-proteasome system, providing a basis for understanding the retinal ciliopathy associated with *TOPORS *mutations.

